# Programming for Stimulation-Induced Transient Nonmotor Psychiatric Symptoms after Bilateral Subthalamic Nucleus Deep Brain Stimulation for Parkinson's Disease

**DOI:** 10.1155/2017/2615619

**Published:** 2017-08-15

**Authors:** Xi Wu, Yiqing Qiu, Keith Simfukwe, Jiali Wang, Jianchun Chen, Xiaowu Hu

**Affiliations:** ^1^Department of Neurosurgery, Second Military Medical University, Changhai Hospital, No. 168 Changhai Road, Yangpu District, Shanghai, China; ^2^Department of Neurosurgery, Changhai Hospital, Second Military Medical University, International College of Exchange, No. 800 Xiangyin Road, Shanghai 200433, China

## Abstract

**Background:**

Stimulation-induced transient nonmotor psychiatric symptoms (STPSs) are side effects following bilateral subthalamic nucleus deep brain stimulation (STN-DBS) in Parkinson's disease (PD) patients. We designed algorithms which (1) determine the electrode contacts that induce STPSs and (2) provide a programming protocol to eliminate STPS and maintain the optimal motor functions. Our objective is to test the effectiveness of these algorithms.

**Materials and Methods:**

454 PD patients who underwent programming sessions after STN-DBS implantations were retrospectively analyzed. Only STPS patients were enrolled. In these patients, the contacts inducing STPS were found and the programming protocol algorithms used.

**Results:**

Eleven patients were diagnosed with STPS. Of these patients, two had four episodes of crying, and two had four episodes of mirthful laughter. In one patient, two episodes of abnormal sense of spatial orientation were observed. Hallucination episodes were observed twice in one patient, while five patients recorded eight episodes of hypomania. There were no statistical differences between the UPDRS-III under the final stimulation parameter (without STPS) and previous optimum UPDRS-III under the STPSs (*p* = 1.000).

**Conclusion:**

The flow diagram used for determining electrode contacts that induce STPS and the programming protocol employed in the treatment of these symptoms are effective.

## 1. Introductions

Subthalamic nucleus deep brain stimulation (STN-DBS) is an effective therapy which ameliorates motor manifestations suffered by patients with idiopathic Parkinson's disease (PD). The hallmark symptoms in PD patients include tremor, rigidity, and bradykinesia. STN-DBS has been documented to be well tolerated by PD patients with marked improvement of motor functions even after over ten-year follow-up [1–3]. The limbic system innervates the limbic part of STN and other anatomic surrounding structures that lay in proximity [4]. As a result, the likelihood of accidental tempering of these nearby anatomical structures during STN-DBS may result in psychiatric symptoms called stimulation-induced transient nonmotor psychiatric symptoms (STPS), which are clinically manifested as depression [5], anxiety [6], apathy [7], explosive-aggressive behavior [8], manic episode [9], mirthful laughter [10], impulse control disorders [11], and so forth. Like other nonmotor functions, in some instances, STPS may have a drastic impact on a patient's life quality of life. [12]. It has been noted that the stimulation parameters which induce STPS are usually higher than normal. Decreasing stimulation intensity on the Implantable Pulsar Generator (IPG) may eliminate STPS. However, patients' motor functions may also be exacerbated. Changhai Hospital Neurosurgery Department conducts more than 160 DBS implantation surgeries every year. Since December 2014, the therapeutic center for Parkinson's disease in Changhai Hospital, Shanghai, China, has designed and implemented (1) an algorithm that identifies specific electrode contacts that induce STPS (2) a programming protocol to eliminate STPS. This study aim is to assess the effectiveness of these algorithms.

## 2. Materials and Methods

### 2.1. Patients

After acquiring approval from the Ethics Committee of Changhai Hospital, we retrospectively analyzed all patients with idiopathic Parkinson's disease who received clinical programming of implanted bilateral STN-DBS in the DBS programming clinic of Changhai Hospital, Shanghai, China, from January 1, 2015, to December 31, 2015. Patients who are being initiated on DBS, as well as those receiving follow-up programming sessions due to various reasons after achieving optimal stimulation parameters, were included in the present study. The UK PD Brain Bank diagnostic criteria were adopted for the diagnosis of idiopathic Parkinson's disease.

### 2.2. Mapping the Active Contact

**Figure 1:**
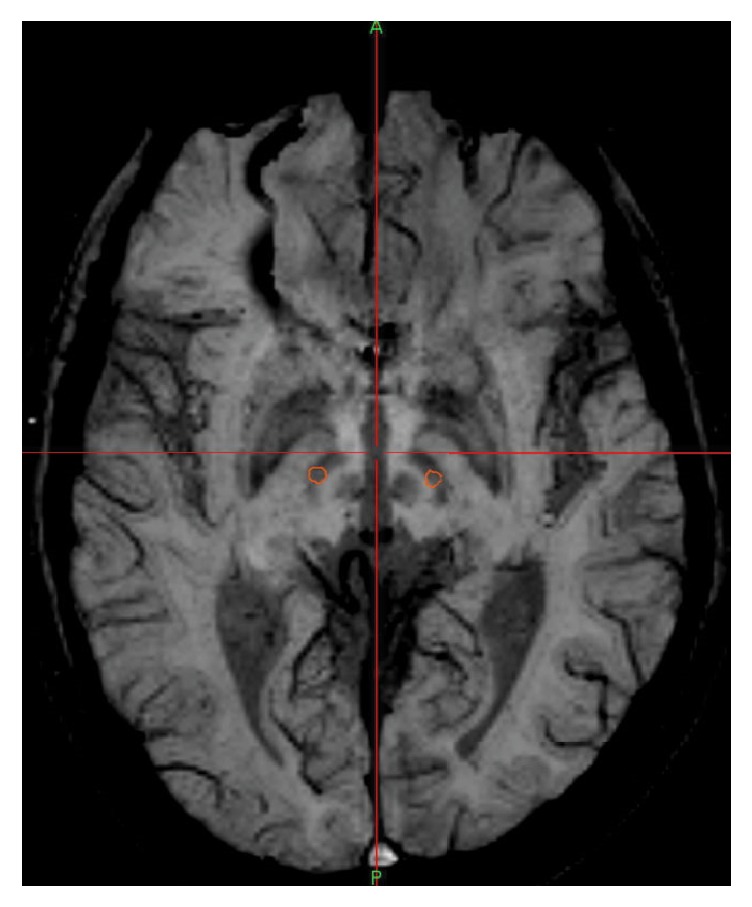
Merging SWI sequence with postoperation CT to determine the electrode position.

If the active contact is not within the STN, the programming protocol may be less effective. Therefore we reviewed intraoperative MRI of all the patients with Leksell G frame and indicator to confirm the electrode location. The Medtronic S7 Neuro Navigation System (Medtronic Navigation, Louisville, USA) was used to merge preoperative 3.0T-MRI images with postoperative CT images. The CT scan images were calibrated at 1 mm thickness and without pneumocranium (Figure 1). It was essential that the active contact center is placed within the boundaries of the STN. Patients with the active contact center outside STN boundary were excluded from this study.

### 2.3. Motor Assessment

Patient's motor function was graded using the Unified Parkinson's Disease Rating Scale Part III (UPDRS-III, 1998 edition). All patients included in this study were assessed preoperatively, postoperatively and then, finally, before and after the occurrence of STPS. All clinicians who administered the Unified Parkinson's Disease Rating Scale in this study are board-certified with the International Parkinson and Movement Disorder Society.

### 2.4. Cognitive and Psychiatric Assessment

All patients with STPS were enrolled in this study. Patients that had any mild psychiatric episodes that occurred more than twice as a result of programming (increasing) the electrode power parameters at home by oneself or caregivers were enrolled in this study. Episodes of acute psychiatric symptoms during or after the clinic programming procedures were also enlisted. Patients with psychiatric symptoms similar to the preoperative ones or had changed medication dose in less than one month were excluded. This was because it would be difficult to confirm whether the symptoms were induced by electric stimulation or not. So, only new postoperative psychiatric symptoms were regarded as suspicious STPS. The litmus test for STPS identification was defined by (1) psychiatric symptoms of patients which improved after decreasing stimulation intensity in 30 minutes; however, this could also confirm that the STPS was induced by stimulation because it occurred at the time when stimulation intensity was increased to improve the patient's motor functions, (2) patients who must have no similar history of psychiatric symptoms and upon clinical programming have shown marked improvement within three months without any change in the medication, and (3) identification of causative STPS active contact.

Cognitive and depressive symptoms were evaluated preoperatively and graded using Unified Parkinson's Disease Rating Scale Parts I and II (UPDRS-I and UPDRS-II), the Mini-Mental State Examination (MMSE), and the 17-item Hamilton Depression Scale (HAM-D-17). Mania type STPS was graded using the Young Mania Rating Scale (YMRS) [13]. The YMRS was calculated using patient collateral history from caregivers and direct observation by the physicians. Patients with STPS while being initiated on IPG were recorded using preoperative evaluation results only.

### 2.5. Assessment of STPS and Recording of Stimulation Parameters

**Figure 2:**
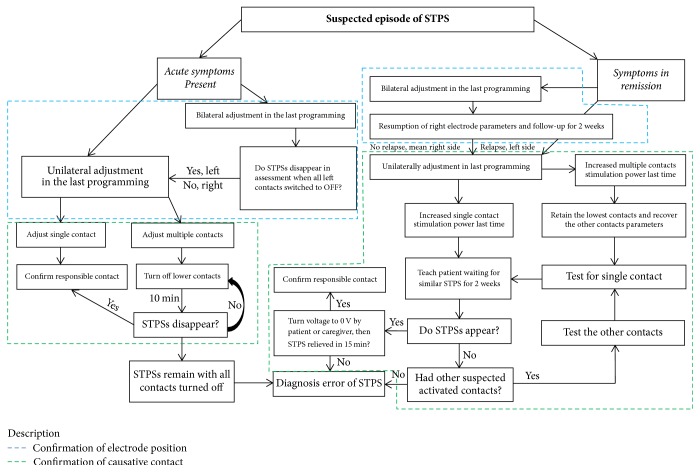
Flow diagram to determine electrode contacts inducing STPS.

**Figure 3:**
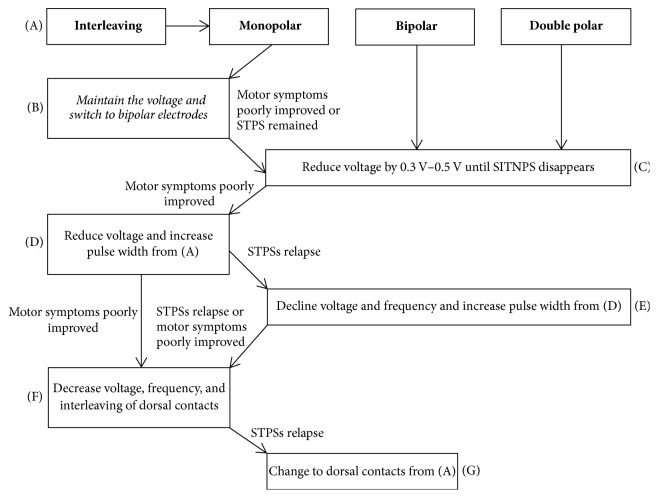
Programming protocol flow diagram (After determining STPS causative active contacts with Figure 2, (A) record the initial parameters and programming according to the stimulation mode; (D) recover the initial parameters and reduce voltage 0.3 V–0.5 V and increase pulse width by 10–20 *μ*s; (E) if STPS relapsed, then reduce voltage 0.1–0.3 V and reduce frequency 10–30 Hz, increase pulse width 10–20 *μ*s, and keep the total electrical energy delivered (TEED) equal to the counterpart of (D), TEED = voltage^2^  × frequency × pulse width/impedance; (F) decrease frequency to 125 Hz and decrease voltage 0.3–0.5 V, and keep the TEED equal or slightly higher to the counterpart of (D) or (E); activate the other dorsal contact with interleaving mode.).

Patient's collateral history regarding unusual psychiatric symptoms recorded in programming sessions was reviewed. Stimulus parameters (active, stimulating contacts, stimulating pattern, voltage/current, pulse width, and frequency) were recorded following the occurrence of STPS. Changes and the final status of stimulation parameters in programming sessions were also recorded. Electrode contacts inducing STPS and programming protocols were determined according to previously established flow diagrams (Figures 2 and 3). The disappearance of STPS was determined by no relapse of similar psychiatric symptoms for three months after programming.

### 2.6. Statistical Analysis

Paired-sample *t*-test was used to examine for changes of variables in UPDRS-III during clinical evaluation. *p* < 0.05 was considered statistically significant.

## 3. Results

### 3.1. Baseline Data

There were three groups of patients enrolled in this study: (1) the first group is comprised of 145 patients undergoing DBS initiation 1 month postoperatively for optimal programming parameter; (2) the second group had 231 patients who underwent DBS less than one year postoperatively; (3) finally, the third group had 166 patients who underwent DBS more than one year postoperatively. There were a total number of 1142 episodes of clinical programming for the purpose of adjusting the stimulation parameters in the second and third groups. There were 20 episodes of STPS that occurred in 11 patients (6 females and 5 males). The mean age at the time of DBS surgery was 63.45 ± 8.27 years. The mean duration between identification of Parkinsonian symptoms and DBS surgery was 11.91 ± 3.33 years. Cognitive functional impairment was excluded in all patients. Only six patients received antidepressant drugs before and after surgery, while the rest of the patients had no obvious depression symptoms. Preoperatively, patients were given an average Levodopa Equivalent Daily Dose (LEDD) of 942.5 ± 232.7 mg. Postoperatively, before the STPS occurred, the LEDD was reduced to 404.5 ± 353.3 mg. By the time programming eliminated STPS at 3 months after programming, the LEDD had further reduced to 284.5 ± 187.8 mg. The UPDRS-III improvement rate between postoperative (med-off time) status and preoperative (med-off time) status was 35.14~72.34% at 3 months after programming. When we compared the baseline data of STPS + patients versus STPS − patients to characterize patients inherent risk factors for STPS, no significant statistical difference was found between groups (see Table 1).

### 3.2. STPS Occurrence

During the programming, 20 episodes of STPS occurred in 11 patients. These psychiatric symptoms consisted of four (04) episodes of crying in two (02) patients, four (04) episodes of inexplicable euphoria or mirthful laughter in two (02) patients, two (02) episodes of spatial disorientation in one patient, two (02) episodes of hallucination in one patient, and eight (08) episodes of hypomania in five (05) patients. In five (05) patients, STPS occurred during titration adjustment for optimal programming parameter in the first year after devices were implanted, while two (02) patients showed psychiatric symptoms two days after being started on IPG and discharged from the hospital. For patients who had implanted devices for more than one year, three (03) developed STPS during programming sessions. Manifestations of patients during STPS episodes were described in Table 2. The stimulation parameters before the onset of STPS were listed in Table 3.

### 3.3. Programming of STPS and Motor Functions

Following the adjustment of stimulation parameters (Table 3), ten patients maintained the improvement of motor functions with psychiatric symptoms eliminated. The adjustments were based on the algorithms as follows: (1) the stimulation voltage decreased while the pulse width increased in 2 patients (numbers 1 and 11); (2) the voltage was maintained while switching to bipolar stimulation in 2 patients (numbers 2 and 7); (3) voltage and frequency were decreased with pulse width increased in 1 patient (number 9); (4) voltage and frequency were decreased while switching to interleaving stimulation in 3 patients (numbers 3, 4, and 10); (5) the activation contacts were replaced with dorsal ones in 2 patients (numbers 5 and 8). One patient (number 6), after multiple programming sessions, developed concurrent motor and nonmotor functions, and UPDRS-III score increased by 2 points under the final stimulation parameters by her choice (see details of the programming duration in Table 4). There were no statistical differences between the UPDRS-III under the final stimulation parameter (without STPSs) and optimum UPDRS-III under the STPSs (26.45 ± 10.59 versus 26.45 ± 10.17, *p* = 1.000).

## 4. Discussion

### 4.1. The Clinical Value of Programming Algorithms for STPS

Since the application of STN-DBS in the treatment of PD patients, there have been sporadic reports [10] noting STPSs, as one of the side effects. The development of these programming algorithms is aiming to reduce the ambiguity in the management of STPS. The ambiguity was on the predisposition of the following. (1) In earlier studies, STPS was implicated with stimulation of the medial and inferior part (limbic part) of STN. However, recent studies show their active contacts located in the dorsolateral (sensorimotor) area of STN [14] which also induces STPS. Because of this reason, the limbic and sensorimotor regions of the STN overlap were greater than what has been previously reported [15]. In this study, we also found that the active contacts which induced STPS were located at a medial and inferior part of the STN in seven (07) patients. In the other four (04) patients, the active contacts were located in the lateral part of STN which also induced STPS. This is similar to what was reported by Abulseoud and colleagues [14]. For this reason, we concluded that the side effects of STPS are difficult to avoid by just implanting contacts into dorsolateral STN. (2) Secondly, under the routine stimulation parameter, the spherical radius of active volume (without contacts volume) could reach to 3 mm. As a result, the limbic part of STN could easily be affected and cause STPS. (3) Thirdly, even in the most experienced PD treatment center could not guarantee that both anticipated contacts location and curative stimulation effect could be satisfied. Meanwhile, STPS could be easily induced when increasing stimulation intensity or testing side effects.

China has the largest number of patients with PD in the world. In recent years, there have been more than 100 centers which carry out STN-DBS surgery for the treatment of PD. As a result, there is a strong likelihood of an increasing number of PD patients suffering STPS after STN-DBS programming. It is therefore very important to rapidly diagnose, locate contacts, and program parameters to eliminate STPS. It is also paramount to maintain the PD patient's optimal motor function according to algorithms set by programming doctors at different levels.

### 4.2. Confirmation of STPS and the Impact Factors

Psychiatric disorders are one of the symptoms manifested in PD patients. However, antiparkinsonism medications also manifest similar symptoms as drug side effects. Levodopa and dopaminergic drugs could induce dopamine dysregulation syndrome, hallucinations and psychosis, mania and hypomania, and impulse control disorders, which were similar to STPS [16]. The treatment of psychiatric symptoms induced by the above drugs is in the reduction of medication in most times. STPS is known to manifest similarly. Therefore the diagnosis of psychiatric symptoms induced by drug side effects or PD should not be misinterpreted for STPS. The diagnosis of STPS should only be made upon confirmation.

Patients in our study had neither any preexisting psychiatric symptoms nor their PD medication altered for than one month. Although preoperative LEDD given to our patients on average was more than the reported average dose before DBS surgery in previous studies [17, 18], the subsequent doses would reduce more than 50% similar to that reported by Jiang and colleagues [17]. Levodopa withdrawal symptoms mainly include apathy and depression. In our study, all 11 patients had no symptoms of apathy and depression. Moreover in the first three-month follow-up after STPS programming, except for the two patients who had STPS at IPG initiation, there was no change in the LEDD in the other patients (Table 1). We, therefore, deduced that the antiparkinsonism medication is not the main factor inducing psychiatric symptoms. Otherwise, some psychiatric symptoms may have reoccurred within the first 3 months of follow-up.

To avoid drugs side effect interference during DBS effect evaluation, all the patients were programming in the state of “off time.” Patients were given daily doses of anti-Parkinson's disease drugs after the final stimulation parameters were set. To achieve the satisfactory therapeutic effects, the motor complications were evaluated during “on time” state. However, the interesting point is that 70% (14/20) STPS occurred in “on time” state or over 1 hour after taking medications (though some patients had not felt medicine effect already); see Table 2. Whether medications for PD work with electrical stimulation to cause psychiatric symptoms or not needs further study. We excluded the effect of PD drugs by maintaining the same dosage that was given on the first occurrence of STPS. So we believe the electrical stimulation should be the main factor of psychiatric symptoms.

To improve the diagnosis of STPS, we developed the algorithm (Figure 2). The causative active contact of patients with acute symptoms of STPS could be easily identified using the flow chart. However, the causative active contact in patients with transient STPS symptoms was more difficult to identify. In the process of locating suspicious contacts in such patients, we do not recommend increasing stimulation intensity to induce STPS to identify contacts. The reason is that (1), theoretically, increasing stimulation intensity of any contact to a certain threshold, the volume of tissue activated (VTA) may affect limbic part of STN and induce STPS. So it may mislead the doctor to regard the contacts which were not the initial responsible contacts. The doctor may even locate wrong responsible electrodes and contacts. (2) If there are multiple active contacts (double polar, interleaving mode) on one side of the electrode, the adjacent contacts have a higher chance to induce STPS (although the ventral contacts are with a higher chance). If randomly increasing stimulation intensity of random contacts to induce STPS, it can also lead to wrong contact. (3) Intentionally induced STPS might cause patients extra distress, leading them to distrust doctors, increased complaints, and decreased satisfaction. Therefore, we recommend the utilization of the algorithm (Figure 2) to locate the contacts carefully and accurately. In our study, the active contacts which induced STPS were identified by using an algorithm (Figure 2) and eliminated by programming in all the 11 patients.

The clinical significance incidence rate of STPS which necessitated programming was about 2%. This was significantly lower as compared to 28.6% in previous studies [14]. This difference may be attributed to the stimulation intensity used and the exclusion criteria we used to enroll patients with STPS symptoms in this study. In this study, the stimulating voltage was rarely more than 3.0 V. However, the stimulating voltage did not exceed 3.3 V.

We noted that time interval for the occurrence STPS fell within 48 hours after new settings of stimulation parameters. Therefore, the psychiatric and psychological status of patients should be thoroughly assessed in MED-ON/STIM-ON after each programming session before patients were discharged. It is also necessary to instruct the family to timely identify the occurrence of STPS, since some symptoms may not be recognized by patients themselves [19].

### 4.3. Principles of STPS Programming

We observed that STPS is likely to develop when programming clinicians increase the voltage or pulse width of the active contact. The increase in active contact voltage or pulse width gives patients a brief false sense of relief masked by improvement in motor functions. Even though the improvement of motor functions are most often minor (1 or 2 point differences in UPDRS-III), STPS set in as residual effects of the increase in voltage. Patients usually find themselves in a quagmire on whether to maintain improved motor functions or settle for removal of STPS and have less appealing motor functions. However, few reports were mentioning how to maintain the optimal improvement for motor functions while avoiding STPS at the same time.

We conducted a literature review using PubMed database of earlier authors who encountered and managed STPS while maintaining optimal motor functions. We designed a programming procedure and protocol to determine the active contacts that induce STPS to rapidly alleviate patients' psychiatric symptoms and maintain improved motor functions.

Previous reports [20] suggest that STPS was associated with high level of voltage. This suggests that the VTA might impact the limbic neuronal networks that induce STPS. They further reported that various programming methods decrease or change the VTA enabling eliminating STPS. These methods include (1) reducing voltage [20], (2) switching to bipolar stimulation [19], and (3) changing dorsal active contacts [9, 21, 22].

Although there are many ways to improve STPS through programming, the order of choice must not be random but with precise principles and in chronological order (Figure 3). These principles include that (1) the solutions for eliminating STPS must be rapid; (2) simple and replicable; (3) and with minimal or no STPS reoccurrence and (4) must preserve optimal motor functions.

There are six methods to adjust the stimulation parameter: (1) bipolar stimulation, (2) reducing voltage by 0.3 V–0.5 V until SITNPS disappears; (3) decreasing of voltage and increasing the pulse width; (4) declining voltage and frequency and increasing pulse width; (5) interleaving of original and dorsal contacts; (6) changing the active contacts to dorsal the position.

#### 4.3.1. Bipolar Stimulation

The first option is bipolar stimulation. We choose to select bipolar stimulation to easily set stimulation parameters and quickly narrow the sphere VTA (Figure 3). This could promote STPS regression and reduce recurrence. However, due to the rapid narrowing of VTA, it often leads to the aggravation of motor functions.

#### 4.3.2. Reducing Voltage

We reduced the voltage on the basis of initial stimulation parameter (with STPS). This adjustment can also quickly reduce the VTA and intensity of the activation domain.

#### 4.3.3. Decreasing of Voltage and Increasing the Pulse Width

The adjustment of programming parameters mentioned above may reduce activation domain [23] while in the meantime declining the stimulating intensity of motor functions-related neural pathways [24]. Therefore a slight increase in the pulse width to maintain stimulation intensity in the narrowed VTA might be needed when the voltage is decreased. Some inexperienced programming physicians may conceive that it is hard to modulate pulse width appropriately. It will be easier to operate according to the formula of total electrical energy delivered (TEED) to calculate how to increase the pulse width according to the voltage reduced (new TEED should be not more than the original one with STPS).

#### 4.3.4. Declining Voltage and Frequency and Increasing Pulse Width

If necessary, we can even adjust the frequency (+5–10 Hz) to fit the change of the voltage and pulse width, to achieve the appropriate stimulation intensity to eliminate STPS while maintaining the improvement of motor functions. In addition to reducing the stimulation frequency to reduce intensity, interleaving technology could be used when adjusting the voltage.

#### 4.3.5. Interleaving of Original and Dorsal Contacts

The adjustment of the pulse width is critical in improving patient's optimal movement symptom after the frequency has reduced to 125 Hz. In the case where the active contact responsible for inducing STPS was ventrally positioned, stimulation parameters remain unaltered. Subsequently, the dorsally located contacts are activated into interleaving stimulation mode. This adjustment has proven to maintain optimal motor function.

#### 4.3.6. Changing the Active Contacts to Dorsal the Position

In isolated patients, motor functions reacted better to stimulation frequency than the later adjustment. As reported, the contacts located anterior, medial, and inferior to STN were easier to precipitate STPS [14]; thus if using the above-mentioned scheme cannot eliminate STPS and achieve optimal improvement of motor functions at the same time, we change the active contact to more dorsal position to acquire a larger range in the adjustment of stimulation parameters. If the inactive contacts were suspended for a long time, curative and side effects might change from previous stimulation record and need retitration which is time-consuming. Also, after the inactivated contacts have been activated, the contact impedance may change with time causing instability of symptom control among patients [25]. We recommend that the interleaving mode generates two isolated VTA: the ventral and dorsal contact. The ventral contact maintains the previous stimulation parameters before the occurrence of STPS. We activate the dorsal contact by gradually increasing the voltage and pulse width to archive optimal motor function [26]. In contrast to the COMPARE trial [27], we have put in consideration preserving patient's motor function while eliminating STPS. Therefore the use of interleaving stimulation was our priority than changing the active contact to dorsal one.

### 4.4. Patient Follow-Up

The impedance of the active contacts reduces over time [25]. Therefore, after STPS programming, patients needed to be followed up for a period to ensure no appearance of similar psychiatric symptoms. STPS was considered eliminated after clinical programming at three (03) months. In the event there was a need to adjust the intensity of stimulation upwards, increasing the width of pulse was given priority. This is because the expansion of the activated domain was not as significant as that of the voltage [28].

### 4.5. Limitations

The limitations of the study are inadequate sample size and limited categories of STPS. Additionally, the flow diagrams adopted in this study are limited to Medtronic Activa PC and RC models. This was a single center study.

## 5. Conclusion

The stimulation contacts in STPS could be determined with use of flow diagram. Appropriate programming could remove STPS while maintaining optimal improvement of motor functions for most patients.

## Figures and Tables

**Table:** (a) Baseline clinical data of patients who developed STPS.

	Age	Gender	Duration (year)	MMSE	HAMD (*α*)	LEDD pre-op (mg)	LEED STPS Emerged	LEDD 3 m post-STPS (mg)	UPDRS I	UPDRS II	UPDRS-III		UPDRS IV
											Pre-op med-off	Pre-op med-on	
PD1	61	M	17	29	19 (14)	1200	1200	550	8	17	47	13	3
PD2	52	M	14	29	10	900	125	125	4	18	41	15	6
PD3	59	F	8	30	21 (16)	768	325	300	7	24	53	26	9
PD4	74	M	7	26	10	563	175	175	6	31	37	24	11
PD5	61	M	8	29	17 (10)	825	825	280	5	11	43	15	10
PD6	58	M	11	26	30 (17)	1100	325	325	10	18	75	42	4
PD7	52	F	15	27	11	963	75	75	2	13	70	34	3
PD8	75	F	13	27	16	1287	300	200	4	20	81	37	8
PD9	72	F	10	28	8	787	250	250	4	13	27	16	4
PD10	64	F	13	26	24 (13)	750	150	150	5	16	51	28	7
PD11	70	F	15	27	30 (16)	1225	700	700	9	22	63	31	10

*α*: post-antidepression treatment; LEDD: Levodopa Equivalent Daily Dose.

**Table:** (b) Compare the baseline data of STPS positive Versus STPS negative patients.

	STPS+	STPS−	*p* value
Patient	11	443^*β*^	
Age	63.45 ± 8.28	62.05 ± 7.78	0.555
Disease duration to DBS	11.91 ± 3.33	9.95 ± 4.03	0.112
LEDD pre-op (mg)	942.55 ± 232.67	836.32 ± 393.79	0.374
UPDRS-III med-off pre-op	53.45 ± 16.94	52.46 ± 14.24	0.819
UPDRS-III med-on pre-op	25.55 ± 9.91	25.80 ± 11.14	0.940
MMSE	27.64 ± 1.43	27.07 ± 2.57	0.463
HAMD before receiving antidepressant drug	17.82 ± 7.85	20.71 ± 6.21	0.130

*β*: 443 patients included three groups: 145 patients initiated the IPG and 99 of them achieved the optimal programming parameters before the study was finished; the 99 patients and other 132 patient who achieved the optimal programming parameters received STN-DBS for less than one year. 166 patients had received STN-DBS for more than one year: 145 + 132 + 166 = 443.

**Table 2:** Clinical manifestations of STPS.

Number	Description of episode					Relationship with medication	Duration
	Times	Chief complaints and symptoms	YMRS	Interval between episode and operation	Time of occurrence		
(1)	1	Inexplicable euphoria (sense of floating)	22	Postoperative 1 month (newly activated)	Day 1 after hospital discharge	Med-on 1.5 h	2 h
	2	Inexplicable euphoria (sense of floating)			Day 2 after hospital discharge	Med-on 1 h	2.5 h

(2)	1	Wail	none	Postoperative 18 months	During programming	MED+ ON	20 min

(3)	1	Hallucination (cloud-like light and shadow)	none	Postoperative 2 months	First night after hospital discharge	Med-on	About 20 min
	2	Hallucination (cloud-like light and shadow)			Second morning after programming	Med-on	30 min

(4)	1	Hypomania (hard to restrain anger)	30	Postoperative 17 months	During programming	Med-off	10 min until adjustment of parameter
	2	Hypomania (sense of anger)	28		During programming	Med-off	10 min until adjustment of parameter

(5)	1	Wail (induced by ordinary conversation)	none	Postoperative 1 month (newly activated)	During programming	Med-off	5 min until adjustment of parameter
	2	Wail (induced by ordinary conversation)			During programming	Med-off	7 min
	3	Wail (induced by talking about the past)			During programming	Med-on	5 min

(6)	1	Hypomania (losing temper to wife)	33	Postoperative 26 months	At home, after programming	Med-on	Improved after 10 min
	2	Hypomania (kicking roadside cars)			At home, after programming	Med-on	2 min, improved 1 min after deactivation

(7)	1	Inexplicable euphoria (laughing foolishly)	20	Postoperative 3 months (during programming)	During programming	1 h after intake	10 h
	2	Inexplicable euphoria (laughing foolishly)			During programming	1.5 h after intake	3 min

(8)	1	Hypomania (lost temper and smashed objects)	34	Postoperative 3 months (during programming)	The 2nd day after hospital discharge	Med-on	2 h
	2	Hypomania (flight of ideas and fidgeting)			The 2nd day after getting home	Med-on	2 h

(9)	1	Hypomania (emotional and quarreling)	34	Postoperative 14 months	Day 6 after hospital discharge; 20 min after raising the voltage of 0.5 V with programming by patients	Med-on 1.5h	1.5 h. Family members restored the stimulation parameter originally set by doctors

(10)	1	Abnormal sense of spatial orientation (unaware of his location when out of clinic)	none	Postoperative 3.5 months (during programming)	During programming	Med-off	10 min, returned clinic for readjustment of parameters
	2	Abnormal sense of spatial orientation			During programming	Med-off	15 min, improved 2 min after parameter adjustment

(11)	1	Hypomania (shouting and fidgeting)	29	Postoperative 2.5 months (during programming)	In the car on his way home after programming	Med-on	1.5 h, improved after readjustment of parameters in the clinic

**Table 3:** The stimulation parameters and STPS.

Number of PD Patients	Status	STPS times	Chief complaints and symptoms of STPS	Stimulation pattern	CV/CC	Intensity (V/mA)	Pulse width (*μ*s)	Frequency (Hz)	Left/right	Cathode	Anode	IPG	Electrode	Active contact location	UPDRS-III score (med off/IPG-on)
(1)	Pre-STPS		IPG-off									Activa RC	3389	Medial STN	
	STPS	1	Inexplicable euphoria	Monopolar	CV	2.5	60	160	Left	1	Case				None^*∗*^
	STPS	2	Inexplicable euphoria	Monopolar	CV	2.2	60	160	Left	1	Case				14
	Final			Monopolar	CV	1.9	70	160	Left	1	Case				14

(2)	Pre-STPS			Monopolar	CV	2.3	80	140	Left	1	Case	Activa RC	3389	Medial part of STN	
				Monopolar	CV	2.5	90	140	Right	9	Case				
	STPS	1	Wail	Monopolar	CV	2.5	90	140	Left	1	Case				13
	Final			Bipolar	CV	2.6	90	140	Left	1	2				13

(3)	Pre-STPS			Monopolar	CV	2.5	60	150	Right	9	Case	Activa RC	3389	Medial Inferior part of STN	
				Monopolar		2.3	60	150	Left	2	Case				
	STPS	1	Hallucination	Monopolar	CV	2.9	70	150	Right	9	Case				25
	STPS	2	Hallucination	Monopolar		2.6	90	130	Right	9	Case				23
	Final			Interleaving	CV	2.5	90	125	Right	2	Case				23
						1.8	70	125		3					

(4)	Pre-STPS			Monopolar	CV	2.3	100	90	Left	1	Case	Activa RC	3389	Inferior part of STN/substantia nigra	
				Monopolar	CV	2.5	110	90	Right	10	Case				
	STPS	1	Hypomania	Double polar	CV	2.4	100	90	Left	0,1	Case				25
	STPS	2	Hypomania	Double polar	CV	2.2	110	85	Left	0,1	Case				25
	Final			Interleaving	CV	2.0	100	90	Left	0	Case				25
						2.3	100	90		1					

(5)	Pre-STPS			IPG off								Activa PC	3389	Lateral part of STN/Close to Zona incerta	
	STPS	1	Wail	Monopolar	CC	1.9	60	140	Right	10	Case				None^*∗*^
	STPS	2	Wail	Monopolar	CC	1.7	60	130	Right	10	Case				13
	STPS	3	Wail	Bipolar	CC	1.8	60	130	Right	10	11				13
	Final			Monopolar	CC	1.9	60	130	Right	11	Case				13

(6)	Pre-STPS			Double-polar	CV	2.5	60	160	Left	0,1	Case	Activa RC	3389	Inferior part of STN/Close to substantia nigra	
				Monopolar	CV	2.7	70	160	Right	9	Case				
	STPS	1	Hypomania	Double polar	CV	2.8	60	160	Left	0,1	Case				40
	STPS	2	Hypomania	Interleaving	CV	2.5	80	125	Left	0	Case				39
						2.8	80	125		1	Case				
	Final			Interleaving	CV	2.5	70	125	Left	0	Case				41
						2.7	80	125		1,2	Case				

(7)	Pre-STPS			Monopolar	CC	2.1	60	145	Left	3	Case	Activa RC	3389	Lateral part of STN/Close to Internal capsule	
				Monopolar	CC	2.0	70	145	Right	10	Case				
	STPS	1	Inexplicable euphoria	Monopolar	CC	2.1	60	145	Left	3	Case				40
	STPS	2	Inexplicable euphoria	Monopolar	CC	1.9	70	145	Left	3	Case				38
	Final			Bipolar	CV	2.3	70	145	Left	3	2				38

(8)	Pre-STPS			Monopolar	CV	2.6	60	150	Left	1	Case	Activa RC	3389	Medial part of STN	
				Monopolar	CV	2.5	60	150	Right	10	Case				
	STPS	1	Hypomania	Monopolar	CV	3.1	60	150	Left	1	Case				40
	STPS	2	Hypomania	Monopolar	CV	2.8	70	145	Left	1	Case				40
	Final			Monopolar	CV	3	60	140	Left	2	case				40

(9)	Pre-STPS			Monopolar	CV	2.7	90	160	Right	9	Case	Activa RC	3389	Lateral part of STN	
				Double-polar	CV	2.5	80	160	Left	2,3	Case				
	STPS	1	Hypomania	Monopolar	CV	3.2	90	160	Right	9	Case				25
	Final			Monopolar	CV	2.9	110	130	Right	9	Case				24

(10)	Pre-STPS			Monopolar	CC	2.0	60	160	Right	9	Case	Activa PC	3389	Medial part of STN	
				Monopolar	CC	2.2	60	160	Left	2	Case				
	STPS	1	Abnormal spatial orientation	Monopolar	CC	2.2	60	160	Right	9	Case				26
	STPS	2	Abnormal spatial orientation	Monopolar	CC	2.0	70	140	Right	9	Case				25
	Final			Interleaving	CV	2.8	60	125	Right	9	Case				25
						2.1	60	125		10	Case				

(11)	Pre-STPS			Monopolar	CV	2.8	70	135	Left	2	Case	Activa RC	3389	Lateral part of STN	
				Monopolar	CV	2.5	60	135	Right	10	Case				
	STPS	1	Hypomania	Monopolar	CV	3.0	70	135	Left	2	Case				34
	Final			Monopolar	CV	2.7	80	135	Left	2	Case				34

CV, constant voltage; CC, constant current.  ^*∗*^The patients with no UPDRS-III score (med-off/IPG-on) because they were in the first programming and have not been evaluated with UPDRS- III score yet.

**Table 4:** Programming procedure of patient number 6.

	Pattern	Voltage (V)	Pulse width (*μ*s)	Frequency (Hz)	Cathode	Anode	UPDRS-III score (med-off/IPG-on)	Chief complaints
Pre-programming	Double polar	2.5	60	160	0,1	Case	41	Spastic pain in the right shoulder
(1)	Double polar	2.8	80	160	0,1	Case	40	STPS occurred
(2)	Interleaving	2.5	80	125	0	Case	39	STPS occurred with hat-like constriction surrounding the head
		2.8	80	125	1	Case		
(3)	Interleaving	2.5	70	125	0	Case	40	Rigidity of neck and posterior extension
		2.8	90	125	1	Case		
(4)	Interleaving	2.5	70	125	0	Case	39	Dizziness and palpitation
		3.1	80	125	1	Case		
(5)	Double polar	2.5	70	130	0,1	Case	40	Fidgeting, feel like preliminary appearance of STPS
(6)	Interleaving	2.5	70	125	0	Case	40	Dizzy and palpitation
		2.7	100	125	1	Case		
(7)	Interleaving	2.5	70	125	0	Case	42	Rigidity of neck and posterior extension with pain in the right shoulder.
		2.8	80	125	1	2		
(8)^*∗*^	Interleaving	2.5	70	125	0	Case	41	Pain in shoulders improved, with no newly developed symptoms
		2.7	80	125	1,2	Case		
(9)	Interleaving	2.5	70	125	0	Case	41	Dizziness
		2.8	80	125	1,2	Case		

^*∗*^The patient chose the 8th programming parameters.

## Additional Points

*Recommendations*. The flow diagrams for patients with other models of IPG should be adjusted accordingly. A meta-analysis could increase the sample size and attain statistical significance.
